# The multifaceted anti-cancer effects of BRAF-inhibitors

**DOI:** 10.18632/oncotarget.27304

**Published:** 2019-11-12

**Authors:** Laura Croce, Francesca Coperchini, Flavia Magri, Luca Chiovato, Mario Rotondi

**Affiliations:** ^1^Istituti Clinici Scientifici Maugeri IRCCS, Unit of Internal Medicine and Endocrinology, Laboratory for Endocrine Disruptors, University of Pavia, Pavia, Italy; ^2^PHD course in Experimental Medicine, University of Pavia, Pavia, Italy; ^3^Department of Internal Medicine and Therapeutics, University of Pavia, Pavia, Italy

**Keywords:** BRAF, BRAF-inhibitors, tumor microenvironment, chemokines, CXCL8

## Abstract

The BRAF gene is commonly involved in normal processes of cell growth and differentiation. The BRAF (V600E) mutation is found in several human cancer, causing an increase of cell proliferation due to a modification of the ERK/MAPK-signal cascade. In particular, BRAFV600E mutation is found in those melanoma or thyroid cancer refractory to the common therapy and with a more aggressive phenotype. BRAF V600E was found to influence the composition of the so-called tumour microenvironment modulating both solid (immune-cell infiltration) and soluble (chemokines) mediators, which balance characterize the ultimate behaviour of the tumour, making it more or less aggressive. In particular, the presence of BRAFV600E mutation would be associated with a change of this balance to a more aggressive phenotype of the tumour and a worse prognosis. The investigation of the possible modulation of those components of tumour microenvironment is nowadays object of several studies as a new potential target therapy in those more complicated cases. At present several clinical trials both in melanoma and thyroid cancer are using BRAF-inhibitors with encouraging results, which are derived also from numerous *in vitro* pre-clinical studies aimed at evaluate the possible modulation of immune-cell density and of specific pro-tumorigenic chemokine secretion (CXCL8 and CCL2) by several BRAF-inhibitors in the context of melanoma and thyroid cancer. This review will encompass *in vitro* and *in vivo* studies which investigated the modulation of the tumour microenvironment by BRAF-inhibitors, highlighting also the most recent clinical trials with a specific focus on melanoma and thyroid cancer.

## The wild type BRAF gene

**Figure 1:**
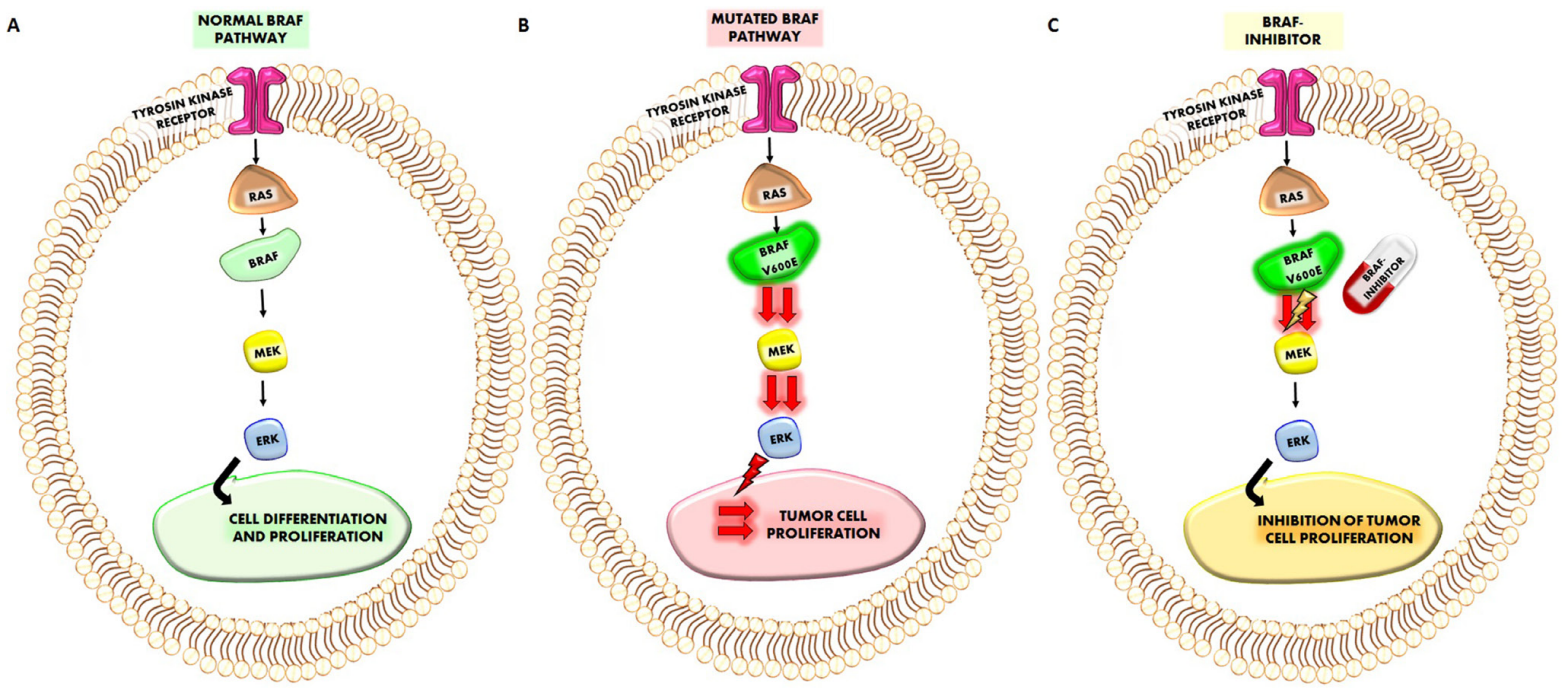
(**A**) Representation of normal BRAF pathway. The sequence of the cascade RAF-BRAF-MEK-ERK starting from the activation of Thyrosin kinase receptor, regulates normal cell differentiation and proliferation. (**B**) When BRAFV600E mutation occurs it will increase the activation of MEK and ERK which induce a more higher cancer cell proliferation. (**C**) The treatment which a given BRAF-inhibitor will reduce the increased activation of MEK (and consequently of ERK) by BRAFV600E mutation reducing also tumor cell proliferation.

The BRAF (v-raf murine sarcoma viral oncogene homolog B1) gene is located on the long arm of chromosome 7 (7q34) and encodes for an 18-exon cytoplasmic protein, a serine/threonine protein kinase (B-Raf) which is recruited to the membrane upon stimulation by growth factors. [1, 2]. The wild type BRAF gene is a downstream effector within the ERK/MAPK signalling pathway, which regulates growth, proliferation, differentiation, and apoptosis in human cells. Chemical signaling through this pathway is essential for normal development before birth [2]. The BRAF gene provides instructions for the transmission of chemical signals from outside the cell to the nucleus, being the MAPK-signaling pathway typically initiated through activation of a membrane tyrosine kinase receptor [3]. This signal, through the activation of RAS, facilitates homo- or hetero-dimerization of wild-type BRAF. Activated BRAF phosphorylates MEK, which, in turn, phosphorylates ERK, resulting in multiple cellular effects such as induction of cell proliferation and survival [3]. (Figure 1A).

## The mutated BRAF gene

When mutated, the *BRAF* gene acquires oncogenic properties. Oncogenes have the potential to promote the transformation of normal to malignant cells [1–3]. Activating mutations of BRAF lead to the constitutive dimerization of the BRAF protein, which in turn activates the RAF-MEK-ERK signalling cascade, thus promoting cell proliferation while inhibiting apoptosis. The final result of this sequence of events is to drive cancer development and growth [3] (Figure 1B). Nowadays, more than 40 BRAF gene mutations have been identified, the majority of which result in changes of the kinase domain and the P-loop of the molecule. These mutated BRAF products actively phosphorylate MEK. Nearly 80% of these genetic alterations correspond to the hotspot T1799A trans-version that causes the V600E activating mutation, which originates from the substitution of valine with glutamic acid at amino acid (aa) 600 [1]. The remaining 20% genetic alterations of BRAF account for a wide range of missense mutations; these reside in the glycines of the G-loop (exon 11) or in the activation segment (exon 15) near the V600. An in-frame fusion of the *AKAP9* gene (exons 1–8) to the *BRAF* gene (exons 9–18), which occurs through a paracentric inversion of chromosome 7, has been preferentially recognised in radiation-induced papillary carcinomas, compared with *BRAF* point mutations [4]. The V600E mutation confers transforming activity to the cells mainly because it mimics the phosphorylation of T599 and/or S602 in the activation segment with the consequence that BRAF remains constitutively active in a RAS independent manner [4]. Mutational studies and crystallography have shown that the BRAFV600E mutation destabilizes the inactive conformation of the enzyme and produces a constitutively active kinase with a 500-fold increased activity [5].

BRAF mutations are associated with a variety of clinical conditions. The cardiac-facies-cutaneous syndrome, a multiple congenital anomaly disorder, is due to BRAF, MEK, ERK or KRAS de novo mutations. Of note common precancerous lesions, such as melanocytic nevi, are characterized by a strikingly high frequency of BRAF-mutations, suggesting that mutational activation of the RAS/RAF/MAPK pathway is a critical step in the initiation of melanocytic neoplasia even if its presence alone is likely not sufficient for melanoma tumorigenesis [6, 7]. Moreover, several malignancies such as melanoma, thyroid carcinoma, and although at lower frequency, colorectal cancer and non-small cell adenocarcinoma of the lung carcinoma may harbor the BRAF V600E mutation. [8–14]. Several data indicate that BRAF-inhibitors block the increase in cell proliferation induced by BRAFV600E mutation (Figure 1C).

The main messages of the present section are:

- *The BRAF gene is involved in proliferation, differentiation, and apoptosis of human cells.*
- *The mutation of the BRAF gene is associated with several human malignancies.*
- *BRAFV600E activating mutation promotes the RAF-MEK-ERK signalling cascade, thus stimulating cell proliferation while inhibiting apoptosis.*

Aim of the present review is to describe currently available data regarding the role of BRAF mutations as well as the effects of BRAF-inhibitors in the tumor micro-environment. Specific focus will be put on melanoma and thyroid cancer, which share the property of being associated with a high prevalence of BRAF mutations.

## The tumor microenvironment in BRAF mutated neoplasia

### BRAF mutation and immune cell composition of the tumor microenvironment

The tumor microenvironment is composed of cells and soluble elements surrounding the tumor [15, 16]. These include fibroblasts, immune cells and cells that comprise blood vessels. It also includes proteins and soluble mediators produced by the cell milieu, which ultimately support the tumor growth [17, 18]. At present, the common belief is that disease progression is promoted by the orchestrated interaction between malignant cells and their surrounding environment [17, 18]. This interaction is in part due to the molecular alterations of the mutated genes, which specifically drive the composition of the tumor microenvironment. In this regard, the BRAF V600E protein mutation was found to be associated with several immune-related alterations, examples of which are available both in melanoma and in differentiated thyroid cancer [1–3, 19–21].

Compared with the non-mutated ones, the microenvironment of BRAF mutated tumors is characterized by a twice as high density of FOXP3+ Regulatory T cells (Tregs), which, by inhibiting the anti-tumor immune response, are associated with a poor prognosis [22]*.* Accordingly, in the early stage of an inducible autochthonous model of mouse melanoma, an accumulation of Tregs occurs in the presence of mutated BRAF [23]. An additional mechanism of immune escape was demonstrated in human melanoma cells, in which BRAF V600E signaling impairs T cell-mediated antitumor responses by increasing the transcription of interleukin 1 alpha (IL-1a) and beta (IL-1b) in cancer-associated fibroblasts resulting in a reduction of their ability to kill melanoma cells [20].

In papillary thyroid cancer, RNA sequencing studies revealed that the presence of BRAF mutation is associated with a reduced expression of immune/inflammatory response genes as compared with wild type-BRAF tumors [24]. Other immunosuppressive molecules, including Human leukocyte antigen G (HLA-G), were also over-expressed in BRAF-mutated tumors [24]. Moreover, the Programmed death-ligand 1 (PDL-1) protein and its mRNA were found to be more abundant in surgical specimens of BRAF-mutated tumors as compared with benign tissue samples. This greater expression of PDL-1 was associated with a denser infiltrate of Treg cells and tumor-associated macrophages [25]. Recently, a genome expression profile analysis of infiltrating immune cells in the microenvironment the BRAF-mutated thyroid cancers showed an over-expression of a panel of genes involved in local immunosuppression processes. These included Cytotoxic T-Lymphocyte Antigen 4 (CTLA4), PDL-1 and HLA-G genes [26].

The main messages of this section are:

- *The presence of the BRAF mutation deeply influences the so called “solid composition” of the tumor microenvironment of both melanoma and thyroid cancer.*
- *BRAF mutation promotes: i) an increase in the density of Tregs, and the secretion of IL-1a and IL-1b in cancer associated fibroblasts (consequently inhibiting the anti-tumor response); ii) a reduction of the expression of some immune/inflammatory response genes (thus inhibiting host defenses); iii) an over-expression of HLA-G and PDL-1 and other immunosuppressive genes (further reducing the immune-system response).*

### BRAF mutation and soluble mediators in the tumor microenvironment

Several studies investigated the chemokine milieu in tumor microenvironment and its influence on the progression and outcome of malignancy [27–30]. Chemokines, an acronym derived from their pro-inflammatory and chemotactic activity [31], and their receptors, do influence immune cell trafficking within the tumor microenvironment, thus eventually promoting or inhibiting tumor progression [30, 32, 33]. Some chemokines, after binding to their receptors expressed on cancer cells, facilitate tumor cell growth by recruiting endothelial cells, by subverting immunologic surveillance, by altering the tumor leukocyte profile in a way that escape from antitumor immune surveillance is favored, and by promoting metastatic processes. Other chemokines play a role against neoplastic progression by increasing leukocyte migration and by inducing long-term anti-tumor immunity [34–37]. The BRAF V600E mutation, by activating the MAPK cascade, stimulates the production of a wide spectrum of chemokines by cancer cells. These oncogene-driven chemokines are responsible for the recruitment of immune cells and for their specific phenotype (i.e. mainly the myeloid lineage) [38].

Experimental studies, both on cancer cell lines and in animal models, provide strong evidence for a correlation between BRAF mutation and altered chemokine secretion in the tumor microenvironment. In human melanoma cells, BRAFV600E was shown to drive the expression of interleukin 6 (IL-6), IL-10 and vascular endothelial growth factor (VEGF), cytokines that, *in vitro*, promote a tolerogenic monocyte-derived dendritic cell (DC) phenotype. This process would theoretically affect the anti-tumor function of T-cell *in vivo* [39, 40]. BRAFV600E was also shown to sustain the constitutive activation of the WNT/β-catenin signaling, which in turn decreases the production of Chemokine C-C motif ligand 4 (CCL4), an important chemokine for the recruitment of dendritic cells. Additionally, BRAFV600E was shown to induce expression of factors such as IL-10 and IL-1α, which can induce tolerogenic forms of dendritic cells and cancer-associated fibroblasts (CAFs), respectively [40]. More recently, the consequences of BRAF mutation on the secretion of CCL2 and chemokine (C-X-C motif) ligand 8 (CXCL8), two chemokines with proven pro-tumorigenic effects, were also evaluated. In a mice model of malignant glioma, the presence of a specific BRAF mutation (KIAA1549) was responsible for a higher expression of CCL2 and of its mRNA [41]. In ovarian cancer cells, the BRAF V600E mutation lead to an increased expression of CXCL8 and of vascular-endothelial-growth-factor A (VEGFA) [42].

The effect of BRAF mutation on the secretion of chemokines was also investigated in human cancer patients. In pediatric patients with Langerhans cell histiocytosis, the BRAF V600E mutation was associated with higher serum levels of the chemokine CCL7, which promotes the metastatic process by inducing epithelial to mesenchymal transition [43]. A significant correlation between elevated levels of C-X-C chemokine receptor 4 (CXCR4) mRNA and a BRAF mutation was found in tissue samples from patients with melanoma [44]. Similarly, CXCR4 levels correlate both with the degree of tumor aggressiveness and with BRAF status in papillary thyroid carcinomas [45]. Husain et al. found that, in tissue samples of anaplastic thyroid carcinoma, the levels of VEGFA, VEGFC and IL-6 increased, and were associated with the expression of BRAF V600E [46].

In view of the above data, it is likely that BRAF mutations largely regulate the expression and/or the secretion of some pro-tumorigenic chemokines in melanoma, thyroid cancer and other types of human tumors. Thus, this mutated oncogene alters the cellular and humoral composition of cancer microenvironment, eventually influencing the biological behavior of malignancy.

The main messages of this section are:

- *The presence of the BRAF mutation also influences the “soluble” composition of the tumor microenvironment (chemokine secretion).*
- *The modulation by BRAF mutation of the secretion of several chemokines (IL-6, IL-10, VEGF, CCL4, IL-1α, CCL2, CXCL8, CCL7) plays a role in: i) influencing the recruitment of immune cells like DC and CAFs (counteracting to the anti-tumor function of T-cells) ii) increasing the metastatic potential of tumor cells and promoting a more aggressive course of the tumor.*

## BRAF-inhibitor drugs

The discovery that most malignant melanomas bear an activating mutation of BRAF [1] lead to the development of BRAF inhibitors (Table 1). These drugs were specifically designed with the aim to insert oncogenic BRAFV600E in the ATP-binding site and trap it in an inactive conformation. [47] The first drug to be tested in humans was Sorafenib, but disappointing results were obtained in melanoma patients, likely due to its greater inhibitory effect on C-RAF rather than BRAF [48]. PLX4720 (Vemurafenib) showed more encouraging results as reported in a Phase II clinical trial, where a favorable response was observed in 48% of BRAF mutated melanoma patients. The corresponding figure in dacarbazine-treated controls was 5%. Thus, the drug was approved by FDA in 2011 and can now be prescribed in patients with metastatic or inoperable melanoma who bear a V600E mutation of the B-RAF gene [49]. Dabrafenib is another BRAF inhibitor tested in a phase III clinical trial with a 50% response rate as compared to a 6% one obtained with dacarbazine [50]. In 2013, the FDA approved Dabrafenib for the treatment of patients with advanced melanomas harboring the BRAFV600E mutation.

**Table 1:** Main BRAF and MEK inhibitors and their characteristics

**Name**	**Formula**	**Target**	**Use**
Sorafenib	BAY43-9006	VEGFR, PDGFR, CRAF, BRAF inhibitor	Kidney, thyroid and liver cancer
Vemurafenib	PLX4720, PLX4032	BRAFV600E inhibitor	Melanoma
Dabrafenib	GSK2118436)	BRAFV600E inhibitor	Melanoma and Non small cell lung cancer
Encorafenib	LGX818	BRAFV600E inhibitor	
Selumetinib	AZD6244	MEK1 and MEK2 inhibitor	
Trametinib	GSK1120212	MEK1 and MEK2 inhibitor	Melanoma
Cobimetinib	XL518, GDC-0973	MEK1 and MEK2 inhibitor	Melanoma (with Vemurafenib)
Binimetinib	(MEK162)	MEK1 and MEK2 inhibitor	Melanoma (with Encorafenib)

In spite of favorable therapeutic effects, these molecules are not free from side effects because, in wild-type BRAF cells, Vemurafenib and Dabrafenib induce a paradoxical activation of the MAP kinase pathway [51]. This event is responsible for common side effects, which include development of kerato-achantomas resulting from the over-expression of the RAS oncogene. Although at lower prevalence, the development of other tumors, including *de novo* melanomas, genital and oral mucosal squamo-cellular cancers and basal-cell carcinoma in patients treated with BRAF inhibitors was also reported [52]. The risk of developing a secondary cancer and mainly the lack of efficacy in BRAF-wild type tumors clearly support the contraindication of both Vemurafenib and Dabrafenib in patients with BRAF-wild type cancers. Moreover, although treatment with Vemurafenib of Dabrafenib produces some clinical benefit in nearly all patients with BRAF mutated melanomas, more than 90% of them develop resistance to these drugs within one year. Thus, the favorable effects on tumor progression-free survival are limited [49]. Based on these considerations, it seemed mandatory to search for new therapeutic strategies targeting the MEK-ERK-RAS pathway. With this aim, two new drugs, Trametinib and Cobimetinib that target MEK downstream from the BRAF in the MAP kinase pathway were developed. These drugs, mainly when combined with Vemurafenib or Dabrafenib, do improve both the overall and the progression-free survival in melanoma patients [53]. Interestingly, combination treatment with the two drugs also reduced cutaneous side effects as compared with the single-drug anti-BRAF therapy [54]. A recent study by Robert et al, reported the results of an extended survival analysis of the two combination trials with Dabrafenib and Trametinib as a first-line therapy, showing that long-term benefit in terms of overall survival can be reached in approximately one third of the patients who had unresectable or metastatic melanoma with a BRAF V600E or V600K mutation [55].

Based on the promising results obtained in melanoma, BRAF-inhibitors have been used also in other BRAF-mutated cancers, with contrasting results. Colorectal cancers that harbour the same BRAF(V600E) mutation are intrinsically resistant to BRAF inhibitors, due to feedback activation of the epidermal growth factor receptor (EGFR), although double or triple combination trials gave some benefit in terms of response rate [56]. Similar results were provided by phase II trials on treatment with dabrafenib of the rare BRAF-mutated non-small-cell lung cancers (NSCLC) in which the BRAF-inhibitor alone gave disappointing results, whereas combination with the MEK-inhibitor Tramentib produced some benefit in terms of overall response rate [57, 58]. Similar evidences were reported in high grade gliomas treated with combined BRAF and MEK inhibitor therapy, although large series studies are still lacking [59].

Up to now, no MAPK/ERK pathway inhibitor drug is approved for the treatment of thyroid cancer [60]. However, Phase I and II clinical trials are ongoing to test the therapeutic potential of this class of drugs, alone or in combination with other pharmacological agents (Table 2). Partial results of these trials are already available. A Phase I study investigated treatment with dabrafenib and lapatinib, the latter being a dual HER2/neu and epidermal-growth factor-receptor (EGFR) inhibitor, in patients with unresectable-radioiodine refractory thyroid cancer (ClinicalTrials.gov Identifier: NCT01947023). In the first 15 enrolled patients, a 60% partial response rate and a median progression-free survival of 15 months was observed. Toxicity was reported to be acceptable [61]. Similar results were obtained in a Phase I clinical trial in which patients with several types of malignancies were treated with Dabrafenib (ClinicalTrials.gov Identifier: NCT00880321). A sub-analysis of the 14 enrolled thyroid cancer patients showed that 29% of them had a partial response, 64% experienced a 10% decrease in tumor burden and 50% had stable disease. The median progression-free survival was 11.3 months [62]*.* Another Phase I study evaluated the ability of Trametinib to induce re-differentiation of radioiodine-refractory BRAF V600E-mutated papillary thyroid carcinoma. Six out of 10 Trametinib-treated patients experienced a restored radioiodine uptake at whole body scan and all of them were then treated with radioiodine. Two patients had a partial response and 4 a stable disease as assessed by radiographic restaging at 3 months. Serum thyroglobulin decreased in 4 out of 6 treated patients [63]. A Phase II clinical trial assessing the effectiveness of Vemurafenib in patients with radioiodine-refractory, BRAFV600E -mutated papillary thyroid cancer reported encouraging results in terms of best overall response, duration of response, and progression-free survival. These favorable results were observed both in previously untreated patients and in those who had received therapy with multi-kinase-inhibitor drugs [64]. The overall toxicity profile was consistent with that reported in melanoma patients being treated with a BRAF inhibitor drug.

**Table 2:** Clinical trials regarding BRAF-inhibitors in Thyroid cancer

Phase	Drug	N. trial	Title	Published Results	Ref
1	Vemurafenib + KTN3379	NCT02456701	Enhancing Radioiodine Incorporation Into BRAF Mutant Thyroid Cancers With the Combination of Vemurafenib and KTN3379	/	/
1	Dabrafenib + Lapatinib	NCT01947023	Dabrafenib and Lapatinib Ditosylate in Treating Patients With Refractory Thyroid Cancer That Cannot Be Removed by Surgery	60% partial response rate, median progression-free survival of 15 months, with acceptable toxicity.	Rothenberg et al. 2015
1	Dabrafenib	NCT00880321	A Phase I Study to Investigate the Safety, Pharmacokinetics, and Pharmacodynamics of GSK2118436 in Subjects With Solid Tumors	29% partial response rate, median progression-free survival of 11 months, with acceptable toxicity.	Falchook et al. 2015
2	Vemurafenib	NCT01286753.	A Study of Vemurafenib (RO5185426) in Participants With Metastatic or Unresectable Papillary Thyroid Cancer Positive for the BRAF V600 Mutation	Increase in best overall response, duration of response, and progression-free survival both in previously untreated and in multikinase-inhibitors treated patients	Brose, et al. 2016
1	Dabrafenib	NCT01534897	Re-differentiation of Radioiodine-Refractory BRAF V600E-mutant Papillary Thyroid Carcinoma With GSK2118436	Among 10 patients with radioiodine-refractory thyroid cancer 6 patients (60%) demonstrated new radioiodine uptake on whole body scan after treatment with dabrafenib. All 6 were treated with 5.5 GBq iodine-131. Two patients had partial responses and 4 patients had stable disease on standard radiographic restaging at 3 months. Thyroglobulin decreased in 4 of 6 treated patients. One patient developed squamous cell carcinoma of the skin. There were no other significant adverse events attributed to dabrafenib.	Rothenberg et al. 2015
2	Vemurafenib	NCT01709292	Vemurafenib Neoadjuvant Trial in Locally Advanced Thyroid Cancer	/	/
2	Dabrafenib + Trametinib	NCT01723202	Dabrafenib With or Without Trametinib in Treating Patients With Recurrent Thyroid Cancer	/	/
2	Trametinib + Dabrafenib	NCT03244956	Efficacy of MEK (Trametinib) and BRAFV600E (Dabrafenib) Inhibitors With Radioactive Iodine (RAI) for the Treatment of Refractory Metastatic Differentiated Thyroid Cancer (MERAIODE)	/	/
1	Trametinib + Pazopanib	NCT01438554	Phase 1 Study of Pazopanib With GSK1120212 in Advanced Solid Tumors, Enriched With Patients With Differentiated Thyroid Cancer, Soft-tissue Sarcoma, and Cholangiocarcinoma	/	/
1	Trametinib + Paclitaxel	NCT03085056	Trametinib in Combination With Paclitaxel in the Treatment of Anaplastic Thyroid Cancer	/	/
2	Trametinib	NCT02152995	Trametinib in Increasing Tumoral Iodine Incorporation in Patients With Recurrent or Metastatic Thyroid Cancer	/	/

The main messages of this section are:

- *BRAF-inhibitor drugs are an effective and relatively safe anti-cancer therapy in patients with melanoma harbouring the BRAF V600E mutation.*
- *Resistance development is still an issue for treated patients and can be only in part overcome by combining BRAF-inhibitors with other classes of anti-cancer drugs, such as MEK-inhibitors and immune check-point inhibitors.*
- *Although no BRAF-inhibitor has been approved for thyroid cancer therapy yet, this class of drugs gave interesting results in Phase I and II clinical trials including patients with thyroid cancer.*
- *BRAF-inhibitors are being tested also in other types of tumors, like colorectal cancer, adenocarcinoma of the lung and glioma, with promising results.*

## BRAF-inhibitor drugs and cancer microenvironment

A further biological effect of BRAF inhibitors is related to their anti-cancer activity in the tumor microenvironment milieu, which, by improving the anti-tumor activity of the patient’s immune system, limits cancer progression. This topic was initially investigated by *in vitro* studies, which were subsequently translated in *in vivo* with promising results.

### *In vitro* experiences

Several *in vitro* studies aimed at evaluating the effect of different BRAF-inhibitors on immune-infiltrating cells (T cells, lymphocytes, dendritic cell) of tumor microenvironment were performed. In 2010, Boni et al., found that treatment of melanoma cells with Vemurafenib (or with other MEK inhibitors) inhibited the MAPK pathway [65]. This inhibition increased the levels of the so called “Melanocyte Differentiation Agents” a class of epitopes that are associated with improved recognition by antigen-specific T lymphocytes [65]. Furthermore, treatment with MEK inhibitors was associated with an impaired T lymphocyte function, whereas T-cell function was preserved after treatment with the BRAF-inhibitor PLX4720. This represents a crucial aspect as, it would indicate that immune evasion of BRAF-mutated melanoma cells may be reversed by a specifically targeted BRAF inhibition without affecting T-cell function [65]. In a BRAF-mutated model of mouse melanoma, the BRAF-inhibitor PLX4720 selectively decreased the number of CD4+Foxp3+ Treg cells and of CD11b+Gr1+ myeloid-derived suppressor cells (MDSC) in the tumor microenvironment. On the other hand, the number of CD8+ effector T cells, which are inversely correlated with tumor growth, was preserved [66]. In a BRAFV600E/PTEN-driven murine model of melanoma, PLX4720 administration increased the expression of CD40 ligand and interferonγ (IFNγ) in intra-tumoral CD4 cells. This increased expression, through the enhancement of T-helper 1 (Th1) effector functions, promotes CD4 cell infiltration and activation which, in turn, leads to tumor regression. In addition, PLX4720 reduced the infiltration of Treg cells and of CD11b(+)/Gr-1(+) myeloid cells, thus further inhibiting tumor growth [67]. Similar effects were also observed in mouse models of thyroid cancer. Indeed, treatment with PLX 4720 alone or in combination with Dasatinib (a BCR/ABL tyrosin-kynase inhibitor) [68], or with anti PD-L1/anti PD-1 antibodies [69] resulted in an increased peri-tumoral infiltration of T cells, B cells and macrophage/monocytes.

**Figure 2:**
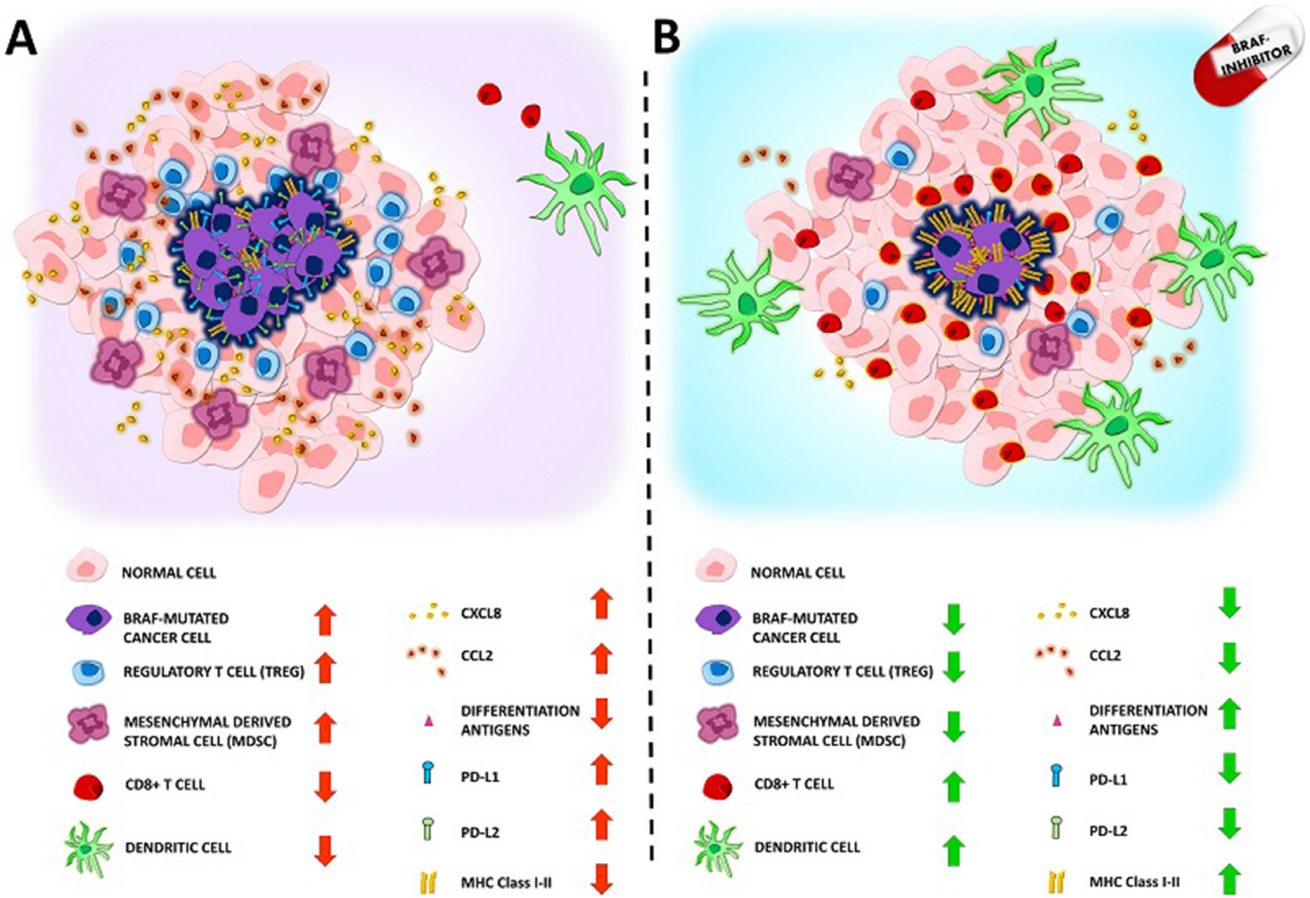
Panel (**A**) Representation of the immunosuppressive microenvironment showed in BRAF mutated cancer. The presence of BRAF V600E mutation favors cancer growth and inhibits the patients immunological response. In detail: a) regulatory T cells (Tregs) infiltration is increased b) Mesenchymal derived stromal cells (MDSC) infiltration is increased c) CD8+ T lymphocytes infiltration is reduced d) Dendritic cells infiltration and function is reduced e) Protumorigenic chemokines CCL2 and CXCL8 secretion is increased f) differentiation agents surface expression is reduced g) PDL1 PDL2 surface expression is increased h) MHC Class I-II surface expression is reduced. Panel (**B**): BRAF mutated cancer immunosuppressive microenvironment can be reverted by treatment with BRAF-inhibitors. In detail: a) regulatory T cells (Tregs) infiltration is reduced b) Mesenchymal derived stromal cells (MDSC) infiltration is reduced c) CD8+ T lymphocytes infiltration is increased d) Dendritic cells infiltration and function is increased e) Protumorigenic chemokines CCL2 and CXCL8 secretion is reduced f) Differentiation agents surface expression is increased g) PDL1 PDL2 surface expression in reduced h) MHC Class I-II surface expression is increased.

The effects of BRAF-inhibitors are not limited to T-cells, but also involve dendritic cells. Indeed, the secretion of tumor-necrosis-factor α (TNF-α) and IL-12 by dendritic cells is inhibited when they are co-cultured with melanoma, cells either BRAF mutated or wild-type. The inhibition was reverted by BRAF or MEK inhibitors, but only when dendritic cells were co-cultured with melanoma cell lines carrying a BRAF V600E mutation [70]. These results fit with the concept that BRAFV600E mutated melanoma cells modulate dendritic cells through the MAPK pathway, because its blockade reverses the suppression of dendritic cell function [70]. In addition, Hayek et al, recently showed that BRAF-inhibitors (Vemurafenib and Dabrafenib) upregulate IL-1β release by mouse and human dendritic cells, thus resulting in enhanced dendritic cell-mediated anti-tumor immune responses [71]. Figure 2 summarize the major effects of BRAFV600E mutation on the solid components of the tumor microenvironment and how the administration of a BRAF-inhibitor would counteract these effects.

Moving from the action of BRAF-inhibitors on immune-infiltrating cells to their effect on cancer cells, it was demonstrated that, in BRAF-mutated melanoma cells, Vemurafenib enhances the presentation of tumor antigens [72]. This effect results from the stimulated secretion of interferons, which in turn induce the expression by neoplastic cells of major histocompatibility complex (MHC) Class I and Class II molecules. In 2010, Salerno et al, investigated the action of PLX 4270 and PLX4032 in thyroid cancer cell lines (bearing either the BRAF mutation or the RET/PTC rearrangement) and in normal thyroid cells. Both compounds inhibited the proliferation of BRAF mutated cell lines, but not of normal thyrocytes [73]. The inhibitory effect was also observed in RET/PTC rearranged cells although at a much higher concentration of the drug. Further evidence for the favorable effect of these drugs in BRAF-mutated thyroid carcinoma cells derives from the observation that treatment with PLX4032 and PLX4720 in BRAF-mutated thyroid carcinoma cells, but not in normal thyroid cells, decreases the phosphorylation of ERK1/2 and MAPK kinase (MEK)1/2. Treatment with PLX4032 and PLX4720 also induces a G1-phase block of the cell cycle and alters the expression of genes involved in the control of G1-S cell-cycle transition [73]. Of note, PLX4720, by downregulating the expression of genes involved in tumor progression, reduced cell proliferation, migration and invasiveness of 8505c, which harbor the BRAF mutation, but not in TPC-1 (wild-type for BRAF) thyroid cancer cells [74].

The anti-cancer effect of BRAF inhibitors was also studied in a orthotopic mouse model of anaplastic thyroid carcinoma, which had been obtained by injecting a thyroid cancer cell line in mice with severe combined immunodeficiency [74]. Compared with sham treated controls, BRAF-mutated tumor xenografts were smaller, less invasive and showed a lower proliferation index when mice received BRAF inhibitors [73, 75]. An even greater anti-tumor effect was observed when treatment with BRAF-inhibitors was combined with: i) anti PD-L1 or anti PD-1 antibodies [69]; ii) the BCR/ABL tyrosin-kynase inhibitor Dasatinib [68]; iii) proteasome inhibitors like Bortezomib [76]. Table 3 summarize pre-clinical studies as well as their main findings concerning this issue.

**Table 3:** Pre-clinical studies regarding the effects on tumor microenvironment of BRAF-inhibitors

Class of Drugs	Effect on microenvironment	Type of cancer	Model	ref
BRAFi, MEKi	Increase of Melanocyte Differentiation Agents	Melanoma	Bioptic samples of treated patients	Boni et al.
BRAFi, MEKi	Reduction of intratumoral Tregs	Melanoma	Braf/Pten mouse model of inducible, autochthonous melanoma on a pure C57BL/6 background	Steinberg et al. Ho et al.
BRAFi, MEKi	Reduction of MDSCs	Melanoma	Braf/Pten mouse model of inducible, autochthonous melanoma on a pure C57BL/6 background	Steinberg et al.
BRAFi, MEKi	Increased dendritic cells activation	Melanoma	co-cultured monocyte-derived human Dendritic cells with melanoma BRAF mutated cell lines	Ott et al. Hayek et al.
BRAFi	Increased intratumoral CD8+ T cells	Thyroid	immunocompetent orthotopic mouse model of V600E BRAF mutated Anaplastic thyroid cancer	Gunda et al, Vanen Borre et al.
		Melanoma	Braf(V600E)-driven mouse melanoma (SM1 and SM1WT1) and melanoma-prone mice	Knight et al.
BRAFi	Increased intratumoral B cells	Thyroid	immunocompetent orthotopic mouse model of V600E BRAF mutated Anaplastic throid cancer	Gunda et al, Vanen Borre et al.
BRAFi	Increased intratumoral Macrophages	Thyroid	immunocompetent orthotopic mouse model of V600E BRAF mutated Anaplastic throid cancer	Gunda et al, Vanen Borre et al.
BRAFi	Preserved normal cell viability	Thyroid	Normal thyrocytes (PC Cl 3)	Salerno et al.
BRAFi	Increased induction of MHC Class I and Class II molecules by IFN	Melanoma	Melanoma cell lines	Sapkota, et al.
BRAFi	CCL2 lowering	Melanoma	Braf(V600E)-driven mouse melanoma (SM1 and SM1WT1) and melanoma-prone mice	Knight et al.
BRAFi	CXCL8 lowering	Thyroid	NHT, 8505C, 8305C, BCPAP cell lines	Coperchini et al.

The main messages of this section are:

- *BRAF-inhibitors modify the composition of tumor microenvironment in experimental settings.*
- *BRAF inhibitors may actually: i) increase the levels of “Melanocyte Differentiation Agents” consequently improving recognition by antigen-specific T lymphocytes ii) decrease the number of Treg and MDSC in the tumor microenvironment iii) increase CD40 ligand and IFNγ expression by intra-tumoral CD4 cells iv) increase peri-tumoral infiltration of T cells, B cells and macrophage/monocytes v) reverse the BRAF-induced suppression of dendritic cell function vi) inhibit proliferation of BRAF mutated cell lines, but not of normal thyrocytes.*
- *The anti-tumor effect of BRAF-inhibitors is even greater when they are used in combination with other classes of anticancer drugs.*

### Translational studies

Evidence that BRAF inhibitors do play a role in determining the specific composition of the tumor microenvironment was also provided by translational studies [77]. These studies were mostly performed on biopsy samples derived from human melanomas. Two independent biopsy series from patients with melanoma who had been treated with Vemurafenib or Dabrafenib + Trametinib showed that, compared with baseline, the expression of melanoma antigens was increased as well the number of infiltrating CD8+ T-cells [78]. A subsequent study showed that treatment with BRAF-inhibitors lead to an increased number of infiltrating CD8+ T-cells. This phenomenon was associated with a reduction in tumor size and an increase in necrotic areas in post-treatment biopsy samples [79]. Additional data [80] showed that in biopsy specimens from BRAF-inhibitors treated patients, the degree of clonality of tumor-infiltrating lymphocytes was greater. This observation implies that infiltrating T-cells are actively proliferating in response to tumor antigens [80]. A neutralization of myeloid-derived suppressor cells in the serum of patients treated with BRAF- inhibitors was also observed. [81]. Table 4 summarizes translational studies on BRAF- inhibitors of as well as their main findings.

**Table 4:** Translational studies demonstrating effects on tumor microenvironment of BRAF and MEK inhibitors

**Class of Drugs**	**Effect on microenvironment**	**Type of cancer**	**Model**	**ref**
BRAFi, MEKi	Increase of Melanocyte Differentiation Agents	Melanoma	Bioptic samples of treated patients	Frederick et al.
BRAFi	Reduction of MDSCs	Melanoma	Serum of treated patients	Schilling et al.
BRAFi	Increased intratumoral CD8+ T cells	Melanoma	Bioptic samples of treated patients	Wilmott et al. Frederick, et al.
BRAFi	CXCL8 lowering	Melanoma	Serum of treated patients	Willmott et al.

The main messages of this section are:

- *Data from preclinical models and from biopsy specimens or blood samples of patients consistently indicate that a targeted therapy with BRAF inhibitors (or with a combination of BRAF and MEK inhibitors) modifies the immune-phenotype of the tumor microenvironment. These effects are mediated by a variety of mechanisms, including increased infiltration and activity of T-cells and enhanced expression and presentation of melanocyte differentiation antigents.*

### Resistance to BRAF inhibitors

It should be highlighted that changes in the composition of tumor microenvironment resulting from treatment with BRAF-inhibitors are not persistent. The escape phenomenon contributes, at least in part, to the high rate of patients who, after an initial response to these drugs, soon develop resistance to therapy and experience clinical progression. Several mechanisms have been suggested as possible cause of resistance to BRAF/MEK inhibitors. These include EGFR and platelet-derived growth factor receptor-β overexpressin [82], increased expression of the gene encoding the COT kinase [83], mutation of downstream MEK1 kinase [84], NRAS mutations [85], increased expression of tyrosine kinases receptor and amplification or alternative splicing of the BRAF gene [86, 87]. Also alterations in the tumor microenvironment seem to have a role in the development of resistance to BRAF/MEK inhibitors. Resistance to BRAF inhibitors was demonstrated in an autochthonous mouse model of melanoma and was associated, in the tumor microenvironment, with the restoration of MDSC, which, previously, had been reduced by treatment with BRAF inhibitors [66]*.* BRAFi-resistant melanomas are also characterized by an increased expression of PD-L1 due to an increased MAPK signaling [78]. Several attempts were made for solving the problem of resistance to BRAF-inhibitors, which mainly involved adding other pharmacological compounds to BRAF-inhibitors. For example, co-treatment with MEK inhibitors partly reversed the expression of PD-L1, due to an increased MAPK signaling, which characterizes melanoma cell lines with acquired resistance to BRAF inhibitors [88]. In BRAF-inhibitors-treated patients who develop drug resistance, the decreased expression of melanoma’s antigens and the reduced number of infiltrating CD8 T-cell parallels the patients’ clinical progression. Again, this phenomenon was largely reversed by a second-line combination of BRAF and MEK inhibitors [78]. Although, the combined use of BRAF and MEK inhibitors reduces the number of patients developing resistance, some patients still develop resistance even to the combined regimen, probably because of an activation of p21-activated kinases (PAKs) [89]. These data suggest that an additional therapeutic benefit in patients with melanoma could derive from the combination of BRAF and MEK-inhibitors with other immunomodulating agents, such as immune check-points inhibitors, agonists of T-cell co-stimulatory receptors, or chemokine/chemokine receptor inhibitors [90]. Indeed, these pre-clinical evidences were recently confirmed by a phase Ib study evaluating the efficacy of the combination of the anti-PD-L1 antibody atezolizumab with vemurafenib alone or in combination with cobimetinib in patients with metastatic melanoma. The results showed that both combination regimens were associated with durable tumor responses, with an overall acceptable toxicity [91]. Furthermore, a randomized phase 2 trial (NCT02130466), comparing advanced melanoma patients receiving dabrafenib+trametinib in combination with the PD-1-blocking antibody pembrolizumab or placebo reported similar results [92]. Although the results of these studies were still inconclusive, ongoing Phase III trials (NCT02967692 and NCT02908672) will probably provide additional information regarding the efficacy of a triple-combination regimen.

The main messages of this section are:

- *Although BRAF and MEK inhibitors provide a significant clinical benefit in melanoma patients, late resistance development remains a major clinical issue.*
- *Changes of the composition of the tumor microenvironment are strictly related with resistance to BRAF inhibitors.*

### Effects of BRAF-inhibitors on chemokines in the tumor microenvironment

The chemokine system is crucially involved in the establishment of tumor microenvironment. A close link also exists between the presence of a BRAF mutation and the secretion of chemokines by resident and infiltrating cells. Several studies, both in melanoma and in thyroid cancer, were aimed at evaluating the potential effect of BRAF-inhibitors on the secretion of chemokines. Because CCL2 has an important role in tumor progression and metastasis, this chemokine was identified as a potential therapeutic target in cancer [93]. Early studies in mice showed that PLX4720 downregulated the expression of the CCL2 gene and of its protein, both in BRAF (V600E)-mutated melanoma xeno-graphs and in *de novo* occurring melanomas. Lowering CCL2 was followed by a reduction of tumor growth [21]. Upon development of resistance to BRAF-inhibitors, human melanoma cell lines further increase their production of CCL2. Similarly, an increase in the serum levels of CCL2 occurs in melanoma patients after extended vemurafenib treatment, and is associated with a poor clinical response [94]. In 2016 Vergani, et al., showed that CCL2 was significantly upregulated both at the transcript and at protein level in BRAF-inhibitor resistant cell lines compared with matched sensitive cells [94]. In addition, the serum levels of CCL2 were higher in patients experiencing a short-term response to BRAF-inhibitor treatment as compared with long-term responders. Taken together these results suggested that CCL2 could be viewed as a potential prognostic factor and an index for resistance to therapy in patients with melanoma [94].

A study in patients with different types of cancer, including melanoma and thyroid cancer, demonstrated that the serum concentrations of another chemokine, CXCL8, were predictive of tumor burden and extent of disease [95]. CXCL8 is the most studied chemokine in human cancer in view of its multiple pro-tumorigenic properties, which span from induction of cell growth to promotion of metastatic processes [96, 97]. Data on this chemokine regard both melanoma and thyroid cancer. Sanmamed et al, reported that patients with metastatic melanoma receiving the PD-1 inhibitor ipilimumab show a decrease or an increase in the serum levels of CXCL8 in relation to a good or poor clinical response, respectively [95]. Based on these findings, CXCL8 received increasing attention in melanoma patients as an important prognostic tool for estimating patient’s tumor burden and disease-free survival [38, 98].

**Figure 3:**
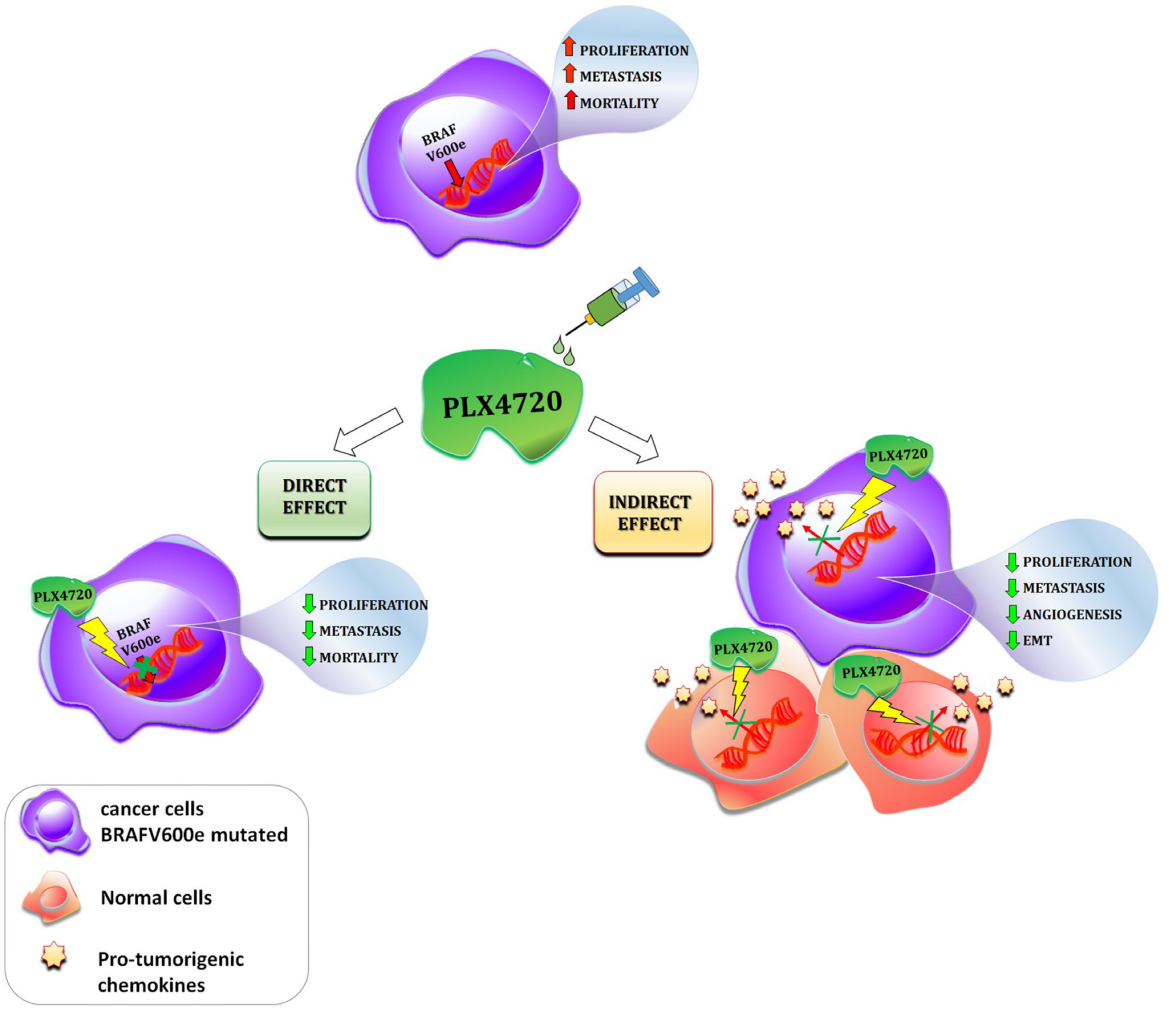
Schematic description of the direct and indirect effects of a given BRAF-inhibitor (PLX4720) in cancer. The presence of the BRAFV600e mutation in cancer cells leads to an increase in cell proliferation, metastasis and patients mortality. The administration of the BRAF-inhibitor PLX4720 exert both direct and indirect effect in cancer. Direct effect: PLX4720 inhibits the molecular pathway switched on by the BRAFV600e mutation, consequently cell proliferation, metastasis and patients mortality are reduced; Indirect effect: PLX4720 inhibits the secretion of pro-tumorigenic chemokines in normal surrounding and cancer cells, which in turn, leads to a reduction of cell proliferation, tumor angiogenesis, EMT and metastatic potential. ****

With specific regards to thyroid cancer, CXCL8 was the first chemokine shown to be secreted by normal human thyroid cells [99–101]. Since then, several studies reported that CXCL8 is also secreted by a wide variety of cancer cell lines including those derived from well-differentiated papillary, medullary, and anaplastic thyroid cancer [102–104]. Experimental evidence demonstrates that thyroid cancer cells produce larger amounts of CXCL8 as compared with normal thyroid cells. In particular, among thyroid cancer cells harboring different oncogenic mutations, those bearing the BRAF V600E mutation secrete the highest amounts of CXCL8 [105]. In the tumor microenvironment, the inhibition of CXCL8 by different compounds (metformin, phenformin, interferons, AICAR,) was proved to exert beneficial anti-tumor effects [100, 106–109]. A recent study by our group showed that the BRAF-inhibitor PLX4720 reduced CXCL8 secretion in several BRAFV600E mutated thyroid cancer cell lines (8505C, 8305C, BCPAP), but not in RET/PTC rearranged ones (TPC1) [110]. Importantly, PLX4720 was able to reduce the migration of thyroid cancer cells, but this effect occurred only in those cells in which PLX4720 also inhibited the secretion of CXCL8 (the BRAF V600E mutated ones). In RET/PTC rearranged cells, PLX4720 did not inhibit CXCL8 secretion and had no effect on cell migration. [110]. These results highlight the concept that PLX4720, by inhibiting the secretion of CXCL8, eventually produces relevant anti-tumor effects. Figure 3 summarizes the possible consequences of BRAF-inhibition on the chemokine system (Figure 3).

Taken together, experimental data in melanoma and in thyroid cancer suggest that BRAF-inhibitors not only exert direct effects on neoplastic cells, but also display an indirect anti-cancer action. The latter is mediated by a reduced secretion of CXCL8 by neoplastic cells, which in turn results in a lower aggressiveness of the tumor. This recently identified inhibition of chemokine secretion by PLX4720 (and potentially by other BRAF inhibitors) implies that our knowledge on the anti-cancer effects in cancer of these drugs is still incomplete. Immunotherapy is currently regarded as the future for cancer cure, and in this view targeting the chemokine/chemokine receptor system might represent a new frontier for the use of BRAF inhibitor drugs.

The main messages of this section are:

- *The modulation by BRAF-inhibitors of chemokines secreted in the tumor microenvironment (in particular of CCL2 and CXCL8) ultimately affects the biological behavior of cancer cells (reducing cell growth and migration).*

## Conclusions

BRAF gene mutations are commonly associated with a more aggressive behaviour of melanoma or thyroid cancer. Data in the literature support the concept that, at least in part, this aggressive behavior results from changes in tumor microenvironment. In this setting, BRAF mutations play a complex role by: i) directly increasing cancer cell proliferation, ii) influencing the immune cell composition of tumor microenvironment, thus creating local immune-suppression, which favors the tumor immune-escape, iii) inducing a greater secretion of pro-tumorigenic chemokines (CXCL8, CCL2), which in turn promotes cancer cell proliferation, angiogenesis and metastatization. In this context, BRAF-inhibitors could represent a useful therapeutic strategy for treatment-refractory patients with either melanoma or thyroid cancer. Indeed, experimental evidence indicates that BRAF-inhibitors directly reduce the proliferation and viability of cancer cells, and indirectly prevent the metastatic process by modulating the chemokine milieu in the tumor microenvironment. Translational studies also support the anti-cancer effect of BRAF inhibitors resulting from changes in the immunophenotype of tumor microenvironment. Clinical trials performed both in melanoma and thyroid cancer patients showed encouraging results when BRAF-inhibitors were tested alone or in combination with other drugs. The development of immunotherapy strategies focused on the tumor microenvironment of BRAF-mutated tumors will hopefully provide new tools for a personalized treatment of patients with melanoma or refractory thyroid cancer.

## Abbreviations

BRAF: v-raf murine sarcoma viral oncogene homolog B1
CAF: cancer-associated fibroblasts
CCL: Chemokine (C-C motif) ligand
CTLA4: Cytotoxic T-Lymphocyte Antigen 4
CXCL: chemokine (C-X-C motif) ligand
CXCR: C-X-C chemokine receptor
DC: dendritic cell
EGFR: epidermal growth factor receptor
FDA: Food and Drug Administration
HLA: Human leukocyte antigen
IFN: interferon
IL: interleukin
MDSC: myeloid-derived suppressor cells
MHC: major histocompatibility complex
PD1: Programmed cell death protein 1
PDL1: Programmed death-ligand 1
PLX4720: Plexxikon compound 7-azaindole derivative
Th1: t-helper 1
TNF: tumor necrosis factor
Treg: Regulatory T cell
VEGF: vascular endothelial growth factor
